# Microseismic Monitoring of CO_2_ Injection at the Penn West Enhanced Oil Recovery Pilot Project, Canada: Implications for Detection of Wellbore Leakage

**DOI:** 10.3390/s130911522

**Published:** 2013-09-02

**Authors:** Patricia Martínez-Garzón, Marco Bohnhoff, Grzegorz Kwiatek, Gonzalo Zambrano-Narváez, Rick Chalaturnyk

**Affiliations:** 1 Helmholtz Centre Potsdam GFZ German Research Centre for Geosciences, Section 3.2 Geomechanics and Rheology, Telegrafenberg, Potsdam 14473, Germany; E-Mails: bohnhoff@gfz-potsdam.de (M.B.); kwiatek@gfz-potsdam.de (G.K.); 2 Institute of Geological Sciences, Free University of Berlin, Berlin 14195, Germany; 3 Department of Civil and Environmental Engineering, University of Alberta, Edmonton, AB T6G 2W2, Canada; E-Mails: gonzalo@ualberta.ca (G.Z.-N.); rc11@ualberta.ca (R.C.)

**Keywords:** CO_2_ injection, passive seismic monitoring, induced seismicity, leakage, continuous seismic recordings

## Abstract

A passive seismic monitoring campaign was carried out in the frame of a CO_2_-Enhanced Oil Recovery (EOR) pilot project in Alberta, Canada. Our analysis focuses on a two-week period during which prominent downhole pressure fluctuations in the reservoir were accompanied by a leakage of CO_2_ and CH_4_ along the monitoring well equipped with an array of short-period borehole geophones. We applied state of the art seismological processing schemes to the continuous seismic waveform recordings. During the analyzed time period we did not find evidence of induced micro-seismicity associated with CO_2_ injection. Instead, we identified signals related to the leakage of CO_2_ and CH_4_, in that seven out of the eight geophones show a clearly elevated noise level framing the onset time of leakage along the monitoring well. Our results confirm that micro-seismic monitoring of reservoir treatment can contribute towards improved reservoir monitoring and leakage detection.

## 1. Introduction

One of the key-challenges in the frame of long-term sequestration of CO_2_ is to deliver appropriate monitoring techniques to document and quantify the safe storage of CO_2_ at selected sites [1,2]. Amongst the approaches to monitor CO_2_ storage, Passive Seismic Monitoring (PSM) can deliver critical information on the effects of pressure perturbation and fracture generation [3,4]. PSM also allows tracing fluid propagation within the reservoir, caprock or along wellbores using locations of small-scale induced earthquakes detected at surface and/or borehole geophones [5].

PSM is a well-established method in both hydrocarbon and geothermal industries, where it is used to monitor reservoir stimulation as well as in fundamental research covering various applications in earthquake seismology. Several studies have used this technique to characterize the treatment of different types of reservoirs [6–12]. Despite the great potential of the method, it is still not systematically applied to the field of CO_2_ storage. However, recent discussions on the feasibility of large-scale CO_2_ storage include the potential risk posed by induced seismicity [13].

There is extensive knowledge supporting the idea that regions with the highest potential for CO_2_ storage are basins with thick sequences of sedimentary rocks [14]. This is the case at the Pembina oil field in Alberta/Canada, where the Cardium Formation (capping siltstones, shales, and sandstones) is confined between Marine Shales and the Blackstone Formation [15]. Recent studies support the view that injection in sedimentary rocks generally tends to be less seismogenic than in crystalline rocks [16]. This observation is consistent with sparse amounts of induced seismic events all being of low magnitude during and after CO_2_ injection in sedimentary formations [17,18]. However, in a recent CO_2_ storage site (In-Salah) many seismic events were induced [19]. In this sense, [20] have shown that the deformation and the geomechanical response of great CO_2_ storage fields can be very different from one site to another. This supports the idea that the few existing case studies cannot be used to generalize the potential for CO_2_ storage sites to generate seismic events. More pilot field studies are needed to derive quantitative statements on the probability of inducing micro-seismicity.

In 2005, the multidisciplinary research pilot project Penn West established by the Alberta Government started injecting supercritical CO_2_ to Enhance the Oil Recovery (EOR) at the Pembina Field [21,22]. At this site, the CO_2_ was injected into the Cardium Formation (1650 m depth) and a percentage of it was systematically released again dissolved in the produced oil. To monitor the CO_2_ injection, a PSM campaign was carried out between 2005 and 2008 using an array of eight three-component borehole geophones. Since the geophones are placed below the uppermost weathering layer and closer to the target reservoir, some of the advantages of using borehole geophones are the substantial improvements of noise conditions with respect to the surface as well as the reduction in the attenuation of the signals.

In this study, we analyse continuous seismic recordings framing a two-week period to investigate whether induced micro-seismicity occurred in the frame of the CO_2_ injection into the reservoir. The selected time period includes a substantial outflow of CO_2_ and CH_4_ (occurring on 1 September 2005 at 09:41 AM) observed at the well-head of the monitoring well where the sensors were deployed. Therefore, we also aim to further investigate whether the outflow resulted in any sort of seismic signatures at the borehole geophones that might serve for an improved detection of along-well gas flow (leakage). Different state-of-the-art seismological analysis methods to detect potential induced seismicity and/or elevated noise levels were performed.

## 2. Data Acquisition

**Figure 1.**
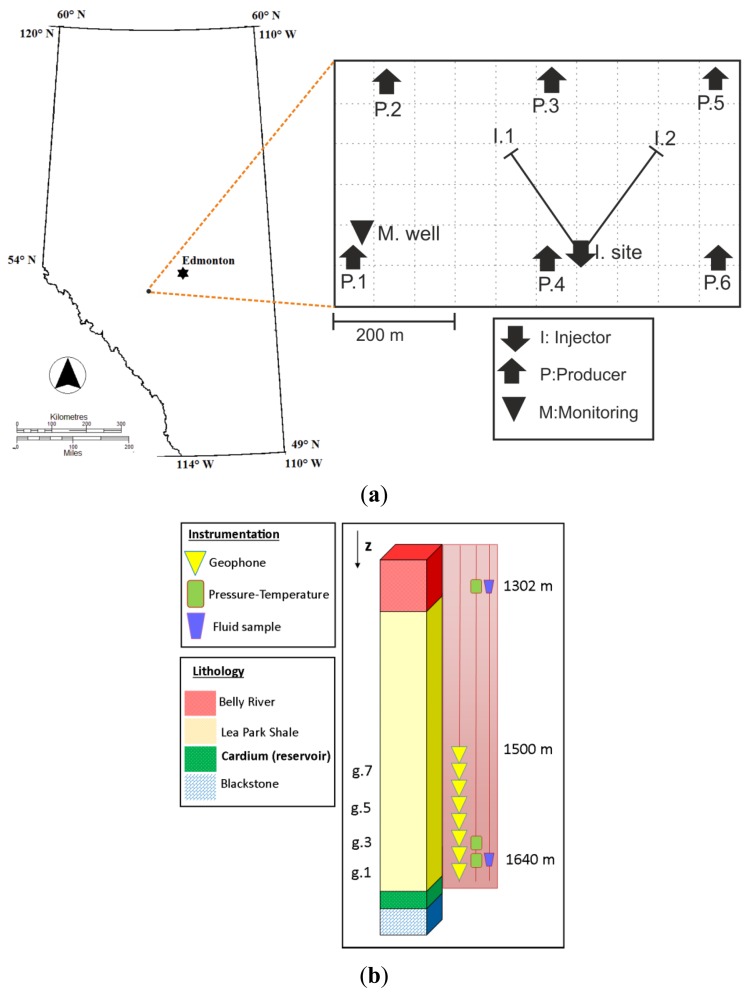
(**a**) Map of the location of the Pembina oil field and location of the monitoring well (triangle) with respect to the injector and the producer wells. P.1–6: Producer wells. I.1, I.2: Injector wells (directional wells); (**b**) Lithological column and instrumentation deployed in the monitoring well. Geophone 1 (g.1) is the deepest sensor, placed at 1640 m. Geophone 8 (g.8) is the shallowest, placed at 1,500 m.

To achieve a comprehensive multi-parameter monitoring of the target reservoir, instrumentation was deployed in a pre-existing vertical production well refurbished as monitoring well. The monitoring well was located at approximately 300 m lateral distance to the nearest injector well I1 (Figure 1a).

The deployed instrumentation at the monitoring well consisted of eight geophones, three pressure-temperature sensors and two fluid-sample sensors (Figure 1b). The instrumentation was attached to production tubing and placed inside the production casing. This procedure is common to reduce the installation damage. To improve the acoustic coupling of the sensors to the formation, cement was retained during the tubing string. However, cementing operations did not proceed as designed and a channel was created in the cement annulus [23]. This fact could affect the coupling of some of the sensors.

The geophones, fabricated from 316 ELC stainless steel are three-component short-period sensors with a natural frequency of 24 Hz and nominal resistance of 12.8 k‖ per axis [24]. They were placed between 1,500 m and 1,640 m depth. Sampling frequency for continuous seismic recordings was set to 1 kHz. Theoretically, they allow to record signals up to 500 Hz. Assuming a conservative average stress drop of 1 MPa, the sensor array should be able to detect nearby micro-seismicity with reasonable high signal-to-noise ratio for M_w_ > −1.5 [25]. This magnitude corresponds to seismic events with source radii of a few meters.

The data from the geophones was analogically acquired and transmitted to the surface. A maximum of four geophone housings could be linked together on a single, 24-conductor (12 pair) stranded copper electrical cable (one pair per each geophone component). Cables were jacketed for safety. This resulted in two electrical cables running to the surface. As the casing was lowered into the well, the geophones were still able to rotate around the vertical axis. For this reason, the horizontal orientation of each sensor is different [26].

## 3. Methods

We have applied different seismological techniques to the continuous seismic recordings to investigate the quality of the data, potential micro-seismic activity and CO_2_ leakage signatures. The applied methodologies are the following:

1. Spectrograms were generated to visually inspect the general frequency content of the waveform recordings. By using spectrograms, micro-seismic events can be identified by short-term amplitude increases in the higher frequency parts (usually >100 Hz, depending on magnitude and hypocentral distance). For this analysis, the waveforms were previously corrected for the baseline shift (detrended) and high-pass filtered (0.8 Hz) to remove potential long-period signals associated with seismic events not recordable by the used instrumentation. Additionally, the data displayed significant noise at 60 Hz and its multiples caused by electrical equipment located nearby. Signals at these frequencies were suppressed by applying two notch filters in the intervals 55‒65 Hz and 115‒125 Hz, respectively. We generated spectrograms for the entire analysed dataset by taking 1 min time-windows of vertical-component waveform data and calculating the short-time Fourier transform of the input signal.
2. We systematically analysed the average noise levels at each individual sensor to determine times of enhanced levels that might be associated with external processes such as e.g., nearby fluid flow. This analysis can also provide information as to the quality of the individual geophones (e.g., due to poor coupling or mechanical dysfunction). Here, we worked with only baseline-corrected data since we were interested in all the frequencies. The noise-level analysis for each individual geophone through the entire two-week data was based on one-minute long subsets.
3. We applied a signal-detection Short Time Average-Long Time Average (STA/LTA) [27] algorithm to identify micro-seismic signatures in the continuous waveform recordings. A STA/LTA trigger detects onset times of characteristic signals (e.g., seismic P and S waves) based on a pre-defined minimum ratio of average absolute amplitudes of two time windows with different length. The STA/LTA ratio will increase once an elastic wave reaches a geophone. When the threshold of the STA/LTA ratio is reached at a particular sensor, the time is saved. For this analysis, the data was processed as for the spectrogram calculation. First, the algorithm was appropriately tuned for this specific dataset. Then, we run the algorithm on the vertical components of the geophones over the entire analysed time period. Finally, a coincidence trigger was applied to the obtained geophone-specific detection lists to select only those seen at a minimum number of four geophones within a given time window (40 ms). To define the time window of the coincidence trigger, a homogeneous velocity model of V_P_ = 3.5 km/s (slightly lower than the estimated V_P_ for the formation in [26]) was used.
4. Lastly, we also looked for potential slow-slip processes included in the data. At reservoir scale, Long-Period and Long-Duration (LPLD) events were found in a multi-stage hydraulic fracturing experiment [28]. The authors described events observed during fracturing periods that have a typical duration of 10–100 s and most of their frequency content is in the 10–80 Hz interval. Recent studies [29] indicated that such events are not necessarily occurring in the frame of reservoir treatment involving hydraulic fracturing. We note that our project was designed to inject large amounts of fluids without causing hydraulic fractures in the target formation, and thus it was not very likely for such signals to occur. Nevertheless, such studies are still quite sparse and it is worth analysing the corresponding frequency band. Note that the frequencies lower than 24 Hz (the natural frequency of the sensors) will be diminished by the transfer function of the sensors. However, since LPLD are reported up to 80 Hz, the available bandwidth to investigate is still sufficient to detect them if they occurred. For this analysis we applied a band-pass filter in the 5‒100 Hz interval. We first stacked the amplitudes for all sensors (vertical components), and then calculated spectrograms of 50 min time-windows by stacking the spectral density of the vertical components.

## 4. Results

Before the described seismological analysis, manual review of the data revealed that for the last 1.5 days of the two-week period none of the channels of g.2, g.4 (partially) and g.6 were functioning. Additionally, it was also noticed that the horizontal components of these sensors display much lower amplitudes than the verticals during the entire analyzed period. Functionality of each geophone is summarized in Table 1.

### 4.1. Spectrograms

**Figure 2.**
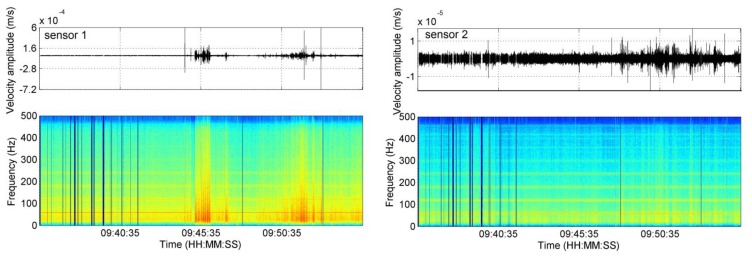

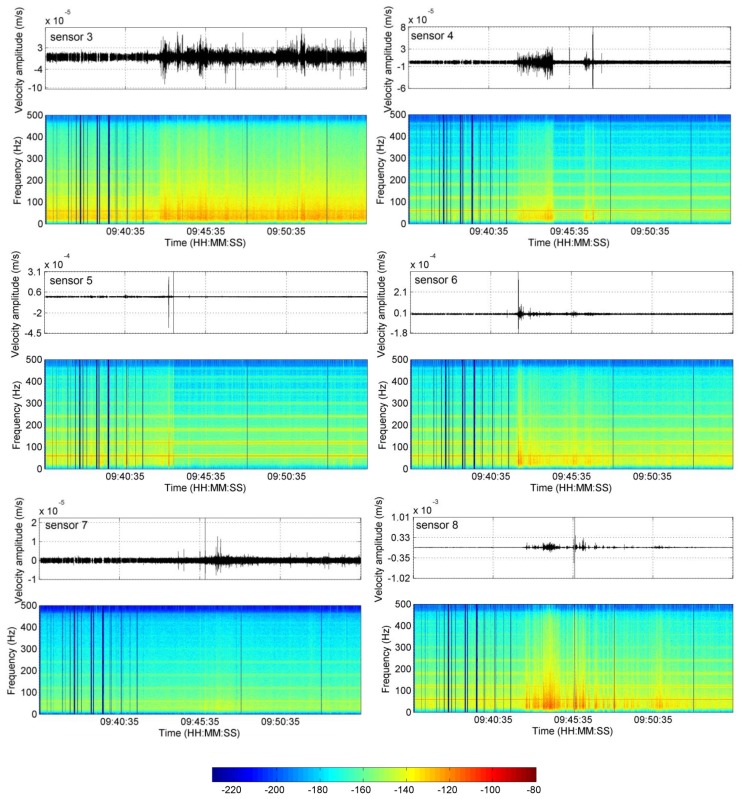
Vertical component waveform recordings and corresponding spectrograms calculated for each geophone framing twenty-minute time-windows around the onset time of the outflow (09:41). The amplitude of each frequency appears color-encoded. g.2 and g.7 have much lower energy recorded than the other geophones. g.1, g.3 and g.8 recorded many sharp spike-signals, although they do not occur at the same time. g.5 and g.6 show spikes with high amplitude, probably triggered internally. Additionally, they still display high electronic noise despite of the notch filter.

The calculated spectrograms show that most of the energy in the recorded time series was transferred in the frequency interval up to 200 Hz (Figure 2). Interestingly, the spectrograms show several short time intervals of elevated energy up to 500 Hz (our Nyquist frequency). Such signals are part of the frequency characteristics of micro-seismic events and thus would need to be checked in detail. However, most of such signals generally do not show any temporal correlation between the individual geophones. This suggests that their origin cannot be external (e.g., related to the injected CO_2_ in the reservoir). For this reason, none of the clear high-amplitude signal seen at the sensors could be related to an induced micro-seismic event occurring off the array.

The spectrograms shown in Figure 2 cover a period of time framing the onset of the outflow (09:41). Many of the sensors show clear changes in the recorded frequency content before and after the onset of the outflow (Figure 2). After 09:41, much more energy is recorded. This energy is especially prominent up to approximately 120 Hz.

Spectrograms were also used to investigate the quality of the coupling of the geophones to the tubing string. A general rule of thumb is, that the better the coupling, the larger is the bandwidth of the transfer function of a borehole geophone. In general, all eight geophones are capable to record also high frequencies indicating a reasonably good coupling to the well-casing (Figure 2). However, the deepest sensor (g.1) and also g.3 recorded overall higher energies.

### 4.2. Noise Analysis

In general, noise amplitudes at the sensors g.1 and g.3 are higher than the noise levels at the other sensors. In addition, g.2 and g.6 recorded significantly lower amplitudes (on average three orders of magnitude less) than any other sensor. Comparing these observations and the manual data review with the field protocols, we found geophones with odd ID numbers shared one common cable and the geophones with even number shared a second one. This resulted in two cables running to the surface. Since common characteristics between the even geophones are found, a second explanation for the low amplitudes recorded would be a higher resistance of the cable resulting in higher attenuation of the signal.

**Figure 3.**
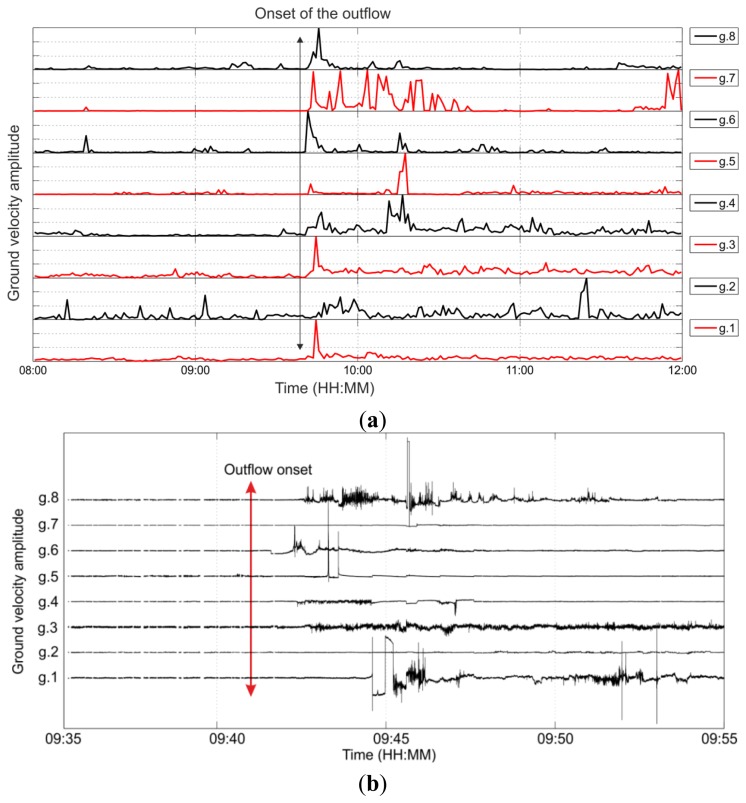
(**a**) Four hours average noise levels at the sensors including the time of the outflow (09:41). Each trace is normalized to its overall maximum; (**b**) Twenty minutes vertical component waveform recordings framing the onset of the outflow. Each trace is normalized to the overall maximum. g: Geophone. Time of the outflow is indicated by the arrow.

**Figure 4.**
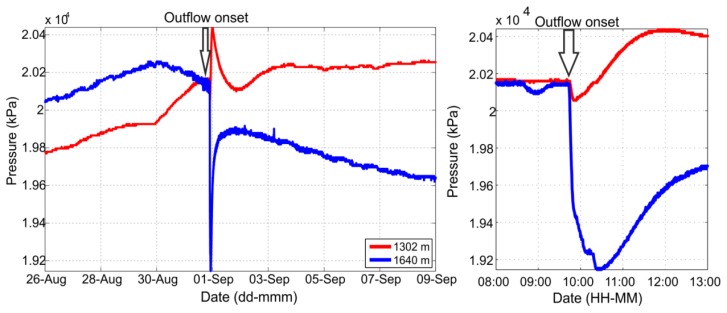
(**Left**): Pressure measured by the sensors inside the observation well during the two-week time period analysed in this study. (**Right**): Zoom on the pressure perturbations at the reported time of the CO_2_ leakage (1 September 2005, 09:41).

During the reported time of the enhanced CO_2_/CH_4_ outflow along the monitoring well (9:41), we find an increase of the noise level for seven out of the eight geophones (Figure 3a). Figure 3b shows twenty-minute waveform recordings framing the onset of the outflow. Clear differences are visible in the waveform signals before and after the onset of the outflow, which might indicate the arrival of the CO_2_/CH_4_ front at the geophone array. The increased noise levels are maintained for the remainder of the monitoring period studied. No clear preference for the outflow detection in terms of channel orientation is found. During the onset of the outflow, most of the sensors present extremely disturbed noise levels but no uniform waveform signatures can be identified. Interestingly, the arrival times of the elevated noise levels are not displaying a linear move out along the array, but in contrast they are time-delayed with no systematic order. To further analyse these signals, we investigated the pressure data measured by the sensors installed at the monitoring well (Figure 4). At the time of the onset of the outflow, the pressure at the sensor at 1,640 m depth decreased by 1 MPa, while the pressure in the sensor installed at 1,300 m increased by 300 kPa. Therefore, there was a dramatic gradient of pressure with both depth and time which subsequently recovered to the respective pre-outflow level after approximately 2 h. The pressure gradient confirms the interpretation of the detected noise level perturbations as a signal related to the CO_2_/CH_4_ migration along the well.

### 4.3. STA/LTA Analysis

**Figure 5.**
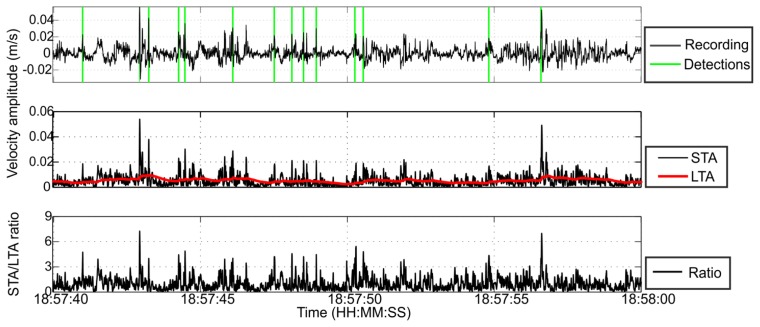
Example of waveform analyzed with STA/LTA. Upper part: filtered recordings for 20 s of data. The green vertical lines are the detections of the STA/LTA. Middle part: STA (black) and LTA (red) functions for the corresponding data period. Lower part: STA/LTA ratio.

Due to the lack of regional seismicity and since no calibration shots were available, we tuned the algorithm parameters based on the accurate detection of several different signals visually identified. Figure 5 shows a waveform data example and corresponding detections of the STA/LTA algorithm.

**Figure 6.**
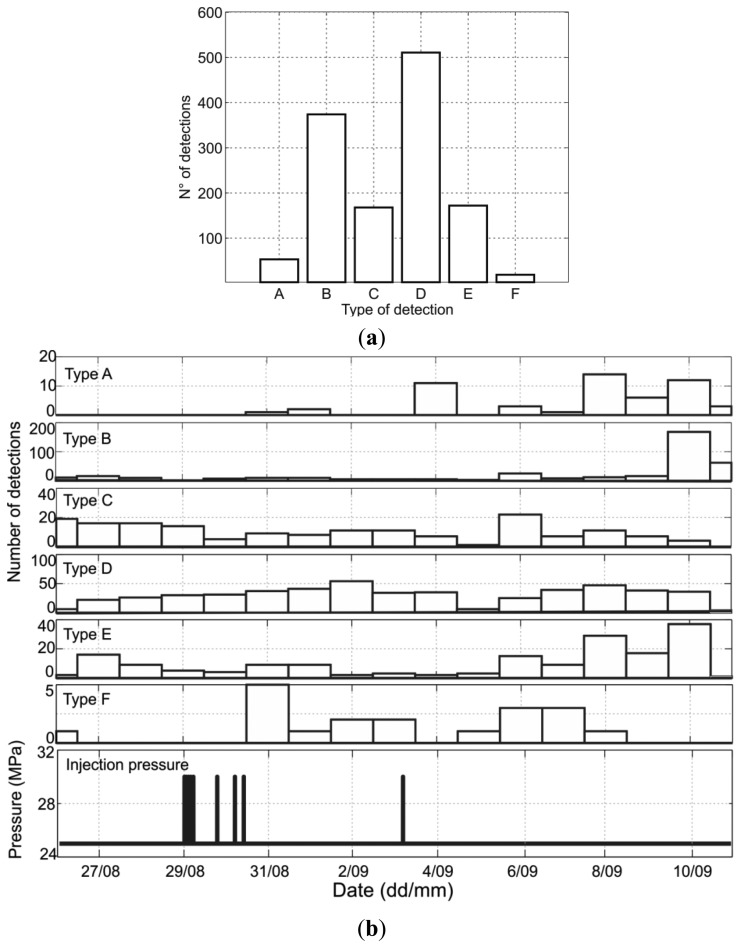
(**a**) Number of STA/LTA detections of each type detected during the entire analyzed period; (**b**) From top to bottom: daily distribution of detection types from A to F and well-head pressure in the Injector well I1 during the two-week period.

The resulting detections of the STA/LTA analysis were visually checked and classified into six different categories (Figure 6a) and example waveform detections are shown in Appendix A.: A-Type detections display large amplitudes at only one geophone, which suggests that the signal was a spike e.g., caused during digitization. B-Type detections typically occur close to the start or end times of periods without recordings (*i.e*., no seismic origin). C-Type detections display larger amplitudes at more than one, but less than four sensors. D-Type detections have extremely low SNR and thus they can be excluded of further analysis. E-Type detections belong to periods when the time series exhibit periodic-electronic signals. These signals are not introduced by the data processing, since we can observe corresponding waveforms also in the raw data. Finally, F-Type detections are signals that have high similarity between the different geophones. Therefore, they have a higher potential to be weak seismic events. However, these signals cannot be associated with typical induced seismicity, since it is not possible to observe P and S phases. For this reason, none of the categories actually represent clear elastic waveforms resulting from failure of rock but rather very local (in part sensor-specific) signals of different origin. Figure 6b shows the daily number of detections for each type. Nearly all A-type and most of the E-Type signals occurred after the onset of the outflow. Since both types of detections might be related with electrical disturbances, they can be seen as an indicator that the leakage of CO_2_/CH_4_ impacted the instrumentation and/or the cables used for the data transmission to the surface. Interestingly, the highest number of Type-F events is registered on 31 August 2005, which is one day after the shut-in of CO_2_ injection into the reservoir (Figure 6b). It is well known that one of the periods with highest likelihood for induced micro-seismicity due to fluid injection is in the shut-in phase. This could be a reason in favour of considering Type-F events as weak induced seismicity. However, the number of events induced is rather small to be able to establish any conclusion in this respect. Additionally, on 3 September 2005, when injection was resumed, electronic spikes and spurious signals increased substantially. Consequently, the last injection might again have damaged the cabling/instrumentation resulting in increasing spurious signals. Alternatively, the recording equipment at the surface might be responsible for generating these signals (through induction or direct impact of the power net).

Since the geophone array was placed on average 1.6 km below the surface, in case of seismic events with their sources at similar depths of the instruments, they might be also recorded on the horizontal components. For this reason, we finally performed an analogous STA/LTA test also on the horizontal components. Once again, the obtained results did not reveal any waveform clearly representing induced micro-seismicity.

### 4.4. Analysis of Low-Frequency Signals

**Figure 7.**
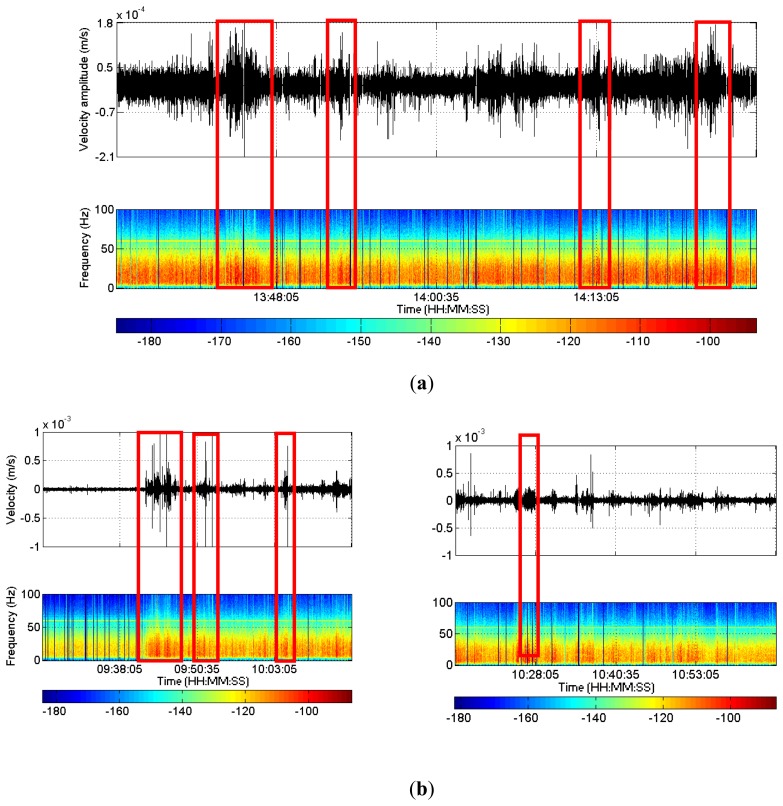
(**a**) Example of signals with high similarity to LPLD events (framed by red rectangles). Upper part: stacking of the amplitudes of the vertical components. The plot has been re-filtered with a band-pass between 5‒40 Hz to reduce electrical noise and better visualize the frequency change. Lower part: Spectrograms for the same time period; (**b**) Similar signals to microseismic events (framed by red rectangles). Upper part: Stack of the amplitudes for the vertical components of every geophone (100 min time window). Lower part: Spectral density stacking for the vertical components of every geophone.

We found potentially relevant signals that display a similar spectral content and duration as the LPLD events (Figure 7a). However, other possible sources for these signals cannot be excluded and, after manual review of the relevant signals in each individual sensor, no coherent signal in several geophones could be identified. Given the unknown orientation of the horizontal components of each geophone, we cannot perform an analogous horizontal stack to check the consistency of these signals in other channels.

The low-frequency data processing pointed our attention towards several signals with similar waveforms of micro-earthquakes, especially following the CO_2_/CH_4_ outflow. Figure 7b shows the waveform data stacking vertical components for a time-window of 100 min and then calculating spectrograms. As pointed out already, after the onset of the outflow (09:41), there is a clear change in the frequency content (see Section 4.1) and several signals with waveforms similar to microseismicity can be identified. However, these signals have lower frequencies than those of typical micro-seismic events, and most of them are only detected in one individual geophone. In consequence, we interpret these signals to be associated with the CO_2_/CH_4_ flow along the monitoring well and passing by the geophones.

## 5. Discussion and Conclusions

In the Pembina oil field there was a small potential for the occurrence of detectable induced seismicity due to its long production record, progressing depletion of the reservoir and the lack of (known) faults in the area. However, induced micro-seismicity might occur due to local pressure perturbations caused by CO_2_ injection into the reservoir and/or leakage of CO_2_ along the monitoring well. In the present study, our primary objective was to investigate potentially occurring CO_2_-induced seismic signatures focusing on a two-week period framing a substantial CO_2_ and CH_4_ leakage along the monitoring well.

The seismological techniques applied to the continuous seismic data recordings did not result in detection of any signal clearly associated with micro-seismic events with source size greater than a few meters. The most promising Type-F events detected with the STA/LTA analysis do not reflect sufficiently strong signals, and therefore it was not possible to determine their source location or to perform any further analysis. The potential LPLD signals identified with the low-frequency analysis cannot be considered as micro-seismicity either since they were detected mainly on one sensor.

As a consequence of the lack of induced seismicity reported, our study is in good agreement with the general view that fluid injection in sedimentary formations tends to trigger less seismicity than injection in crystalline rocks. This statement, of course, is subjected to the volume of injected CO_2_.

The most striking observation from our analysis is that we clearly identified signals related to the outflow of CO_2_/CH_4_ in that seven out of the eight geophones show an elevated noise level and complex signals during the reported onset. The fact that these signals are not occurring simultaneously leaves open several questions related to the actual processes triggering these signals. Since no preference in terms of channel detection was found for the elevated noise, and given the observed complexity of the signals, the origin of the elevated noise could be partially electronic disturbances introduced by the front of CO_2_/CH_4_ arriving to the sensors and damaging certain components.

In some studies, seismic signals associated with the drastic volume increase of CO_2_ during its phase change from supercritical to gaseous have been reported [17,30]. For the Penn West Pilot Project at the Pembina Field, the thermal gradient and reservoir pressure were 2.9 °C/100 m and 2.10 MPa, respectively [31], and therefore it is reasonable to believe that the phase change of the CO_2_ and the resulting elastic waves would occur at depth levels around 1,000 ± 100 m. Since in our case the geophone array was placed much deeper (1,500–1,640 m) and given the small energy of such signals, the current location of the monitoring equipment would not allow detecting them, although they might have occurred in the context of the outflow. This conclusion, however, is restricted to CO_2_, while any similar behaviour for the CH_4_ would need to be evaluated in detail separately.

Due to the CO_2_/CH_4_ leakage reported along the observation well, the data from our borehole geophone array provide valuable hints about processes related to the migration of CO_2_. This case study is a good example to illustrate the importance of performing an appropriate deployment of the instrumentation and a good preparation of the data acquisition system in order to obtain reliable and correct data for the reservoir monitoring.

## Figures and Tables

**Table 1.** Summary of geophone status obtained from spectrograms, noise level analysis and manual review of the data.

**Geophone ID**	**Depth (m)**	**Geophone Functionality**
8	1,500	Correct
7	1,520	Horizontal component ‘x’ not recording
6	1,540	Horizontal components ‘x’ and ‘y’ not recording
5	1,560	Correct
4	1,580	Correct during certain periods
3	1,600	Correct. High noise level (average amplitudes)
2	1,620	Horizontal components ‘x’ and ‘y’ not recording
1	1,640	Correct. High noise level (average amplitudes)

**Figure A1.**
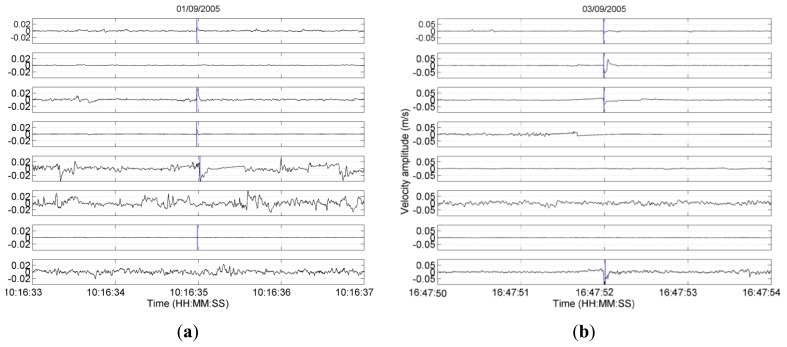

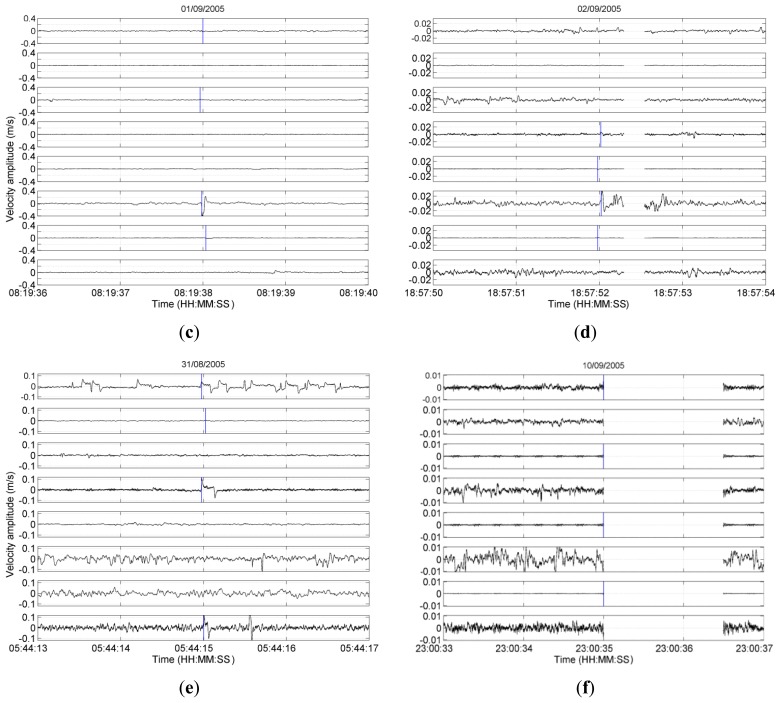
Examples of signals identified by the STA/LTA observed during the two-week time period analysed in this study. Each subfigure shows the vertical components of geophones 1 to 8 from bottom to up. Date of the detection appears in the top of each plot (dd/mm/yyyy). Each trace is normalized by the maximum of and interval which covers 0.1 s before and after the detection. (**a**–**c**): Examples of detections from Type-F events. (**d**): Type-A event with the largest amplitude registered in geophone g.3. (**e**) Type-B event at the edge of a period without recording (typical recording for the last days of the two-week period). (**f**) Type-E event in which periodic signals appear in g.8.
